# Local versus global aortic pulse wave velocity in early atherosclerosis: An animal study in ApoE^-/-^-mice using ultrahigh field MRI

**DOI:** 10.1371/journal.pone.0171603

**Published:** 2017-02-16

**Authors:** Alexander Gotschy, Wolfgang R. Bauer, Patrick Winter, Peter Nordbeck, Eberhard Rommel, Peter M. Jakob, Volker Herold

**Affiliations:** 1 Department of Experimental Physics V, University of Würzburg, Würzburg, Germany; 2 Department of Cardiology, University Heart Center, University Hospital Zurich, Zurich, Switzerland; 3 Institute for Biomedical Engineering, University and ETH Zurich, Zurich, Switzerland; 4 Department of Internal Medicine I, University Hospital Würzburg, Würzburg, Germany; 5 Comprehensive Heart Failure Center / Deutsches Zentrum für Herzinsuffizienz, University of Würzburg, Würzburg, Germany; Ludwig-Maximilians-Universitat Munchen, GERMANY

## Abstract

Increased aortic stiffness is known to be associated with atherosclerosis and has a predictive value for cardiovascular events. This study aims to investigate the local distribution of early arterial stiffening due to initial atherosclerotic lesions. Therefore, global and local pulse wave velocity (PWV) were measured in ApoE^-/-^ and wild type (WT) mice using ultrahigh field MRI. For quantification of global aortic stiffness, a new multi-point transit-time (TT) method was implemented and validated to determine the global PWV in the murine aorta. Local aortic stiffness was measured by assessing the local PWV in the upper abdominal aorta, using the flow/area (QA) method. Significant differences between age matched ApoE^-/-^ and WT mice were determined for global and local PWV measurements (global PWV: ApoE^-/-^: 2.7±0.2m/s vs WT: 2.1±0.2m/s, P<0.03; local PWV: ApoE^-/-^: 2.9±0.2m/s vs WT: 2.2±0.2m/s, P<0.03). Within the WT mouse group, the global PWV correlated well with the local PWV in the upper abdominal aorta (R^2^ = 0.75, *P*<0.01), implying a widely uniform arterial elasticity. In ApoE^-/-^ animals, however, no significant correlation between individual local and global PWV was present (R^2^ = 0.07, P = 0.53), implying a heterogeneous distribution of vascular stiffening in early atherosclerosis. The assessment of global PWV using the new multi-point TT measurement technique was validated against a pressure wire measurement in a vessel phantom and showed excellent agreement. The experimental results demonstrate that vascular stiffening caused by early atherosclerosis is unequally distributed over the length of large vessels. This finding implies that assessing heterogeneity of arterial stiffness by multiple local measurements of PWV might be more sensitive than global PWV to identify early atherosclerotic lesions.

## Introduction

Arterial stiffening and accompanying elevated pulse wave velocities are an inherent characteristic of vascular aging as well as of atherosclerosis [1, 2]. Moreover, pulse wave velocity (PWV) has proven to be an independent predictor of cardiovascular risk and mortality as higher aortic stiffness, assessed by PWV, was shown to be associated with increased incidence of cardiovascular events [3, 4]. To determine the global PWV, the transit-time (TT) technique, e.g. measurement of the carotid-femoral PWV has emerged as the gold standard in clinical practice. In this approach, the pulse wave arrival times are determined at two separated sites along the vessel. Global PWV can then be computed as the distance between the two sites over the delay between the pulse wave arrival times [5, 6].

The examination of small animal models of human cardiovascular physiology adds to the understanding of the origin, progression, and the treatment of cardiovascular disease (CVD). Apolipoprotein E-deficient (ApoE^-/-^) mice are considered to be an important model of atherosclerosis, since they develop atherosclerotic lesions of morphology similar to those observed in humans [7–9].

While cardiovascular MRI has been extensively used to characterize morphological and biochemical processes during the progression of murine atherosclerosis [10–15], only few studies investigated the evolution of arterial stiffening caused by atherogenesis. Those studies were limited to echocardiographic [16, 17] or MR-based [18, 19] TT measurements of global PWV corresponding to the mean stiffness of the vessel and thus leaving a gap of knowledge regarding the spatial distribution of arterial stiffening along the vessel.

Recently, non-invasive techniques for the determination of the local PWV, like the flow/area (QA)-method, have become available and have shown to provide additional information regarding the arterial elasticity at a specific site of the vessel [20, 21]. In ApoE^-/-^ mice, it has lately been shown that local PWV, assessed by MRI, is elevated even before a morphological thickening of the vessel wall occurs [22].

In the present work, local PWV in the abdominal aorta and global PWV were comparatively assessed in WT and ApoE^-/-^ mice with early atherosclerotic lesions, to investigate the local distribution of early arterial stiffening in the aorta. To measure global PWV in mice, a recent publication demonstrated the feasibility of a two point TT-technique, using phase-contrast (PC) Cine-MR imaging [19]. We advanced this method to a multi-point TT-technique by transferring the principles of the TT method to in-plane flow encoded MRI. All local and global PWV-measurement techniques were validated in a vessel wall phantom prior to in-vivo measurements.

## Materials and methods

All MR experiments were performed on a Bruker AVANCE 750 (Bruker Biospin, Rheinstetten, Germany) NMR spectrometer with a vertical main magnetic field of 17.6 T and a bore size of 89 mm.

The system was equipped with a self-shielded gradient insert with an inner diameter of 40 mm capable of 1000 mT/m maximum gradient strength. Two laboratory built birdcage resonators with an inner diameter of 20 mm and 25 mm were used for RF transmission and reception in mice with a weight of less than 24 g and above, respectively. Cardiac and respiratory trigger signals were generated using a pressure sensitive balloon by detecting chest wall motion. The pressure signal was transformed into an electrical signal outside the gradient coil and was post processed in real-time with a homebuilt amplification unit. This mechanical trigger technique allowed for a complete suppression of interferences with the rapidly switching strong gradient fields.

### Multi-point transit-time method for measuring the global PWV

**Fig 1:**
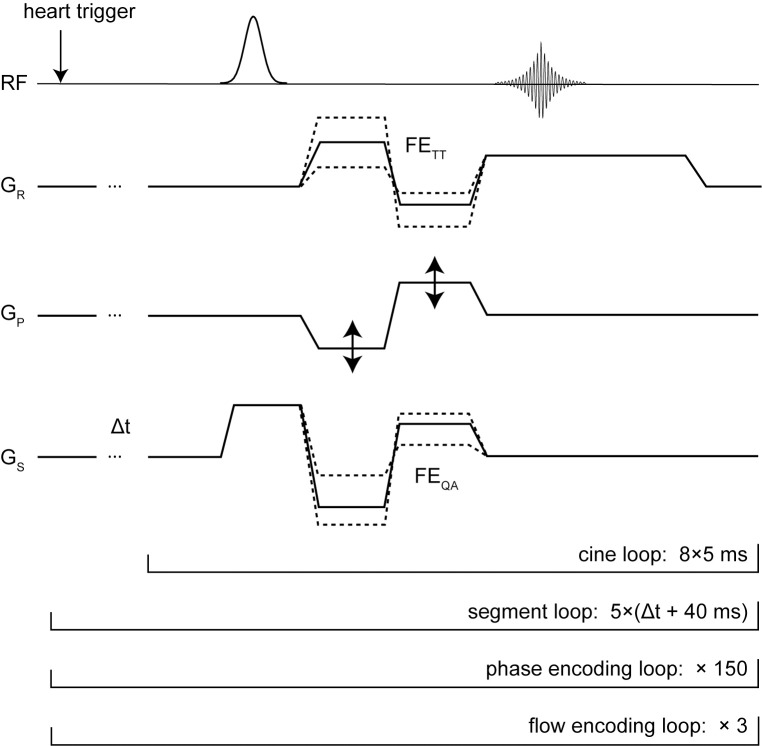
Phase-Contrast-Cine pulse sequence. Phase-Contrast-Cine pulse sequence to measure global and local pulse wave velocities (PWV). The sequence was repeated five times (segment loop) starting with a variable delay Δt = (0ms, 1ms, 2ms, 3ms, 4ms) thus yielding to an effective temporal resolution of 1000 frames per second. Further abbreviations: RF: radio frequency transmission; G_P_: phase encoding gradient; G_R_: frequency encoding gradient; G_S_: slice encoding gradient; FE_QA_: axial flow encoding for the local PWV measurement; FE_TT_: 1D in-plane flow encoding for the global PWV measurement.

To determine the global PWV with the multi-point TT method, a 2D-PC-CINE data set with 1D axial velocity encoding parallel to the flow direction was acquired. The basic imaging sequence consisted of a 2D fast low angle shot (FLASH) sequence with velocity compensated gradients for all three gradient directions. Motion encoding was performed using bipolar gradients along the frequency-encoding direction (v_enc_ = 1.7 m/s) in two additional symmetric flow encoding steps, as depicted in Fig 1. The gradient pulses for motion compensation were added to the bipolar gradients for motion encoding to minimize the total echo-time.

For *in vivo* experiments the imaging slice was oriented to align the flow direction in the descending aorta with the frequency encoding direction. Since the stability of the cardiac heart rate during the measurement is crucial to provide sufficient accuracy of the PWV calculation, the total measurement time had to be minimized. Therefore only a one-dimensional flow-encoding scheme was applied which restricted the PWV evaluation to the linear section of the descending aorta. To increase the temporal resolution from 5 ms (TR) to 1 ms, a total of five interleaved, time-delayed imaging experiments were performed starting with variable delays of Δt = (0,1,2,3,4) ms [20].

### QA Method for measuring the local PWV

Assuming that the forward traveling pulse wave is free of any reflected backward traveling pulse, in the early systole, the local PWV can be estimated as a function of the blood volume flow Q(t) and the cross sectional area A(t). Using the expression for the characteristic impedance and the wave equation for inviscid flow, it can be shown that the local PWV can be derived with the following equation [21]:

$$PWV=\frac{dQ}{dA}$$

The time course of local cross-sectional area and flow were acquired simultaneously using a 2D-PC-CINE-pulse-sequence with 1D velocity encoding in through plane direction [20, 22].

### Phantom experiments

**Fig 2:**
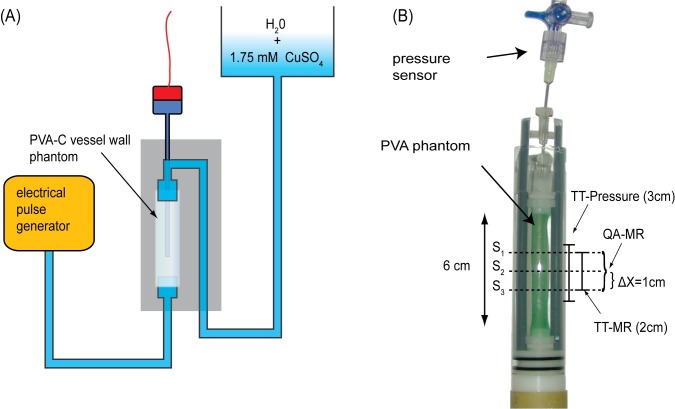
Phantom experimental setup. Panel **(A)** shows a schematic depiction of the PVA-C phantom with electrical pulse generator and pressure sensor. The pulse wave velocity phantom actually mounted is shown in **(B)**. The sites for the transit-time pressure measurements and the transit-time MR measurements as well as the measurement slices for the QA-measurement are marked.

Local and global PWV measurement techniques were compared and validated in a vessel wall phantom made of polyvinyl alcohol cryogel (PVA-C) [23]. The experimental setup for the MR-measurements was chosen accordant to the in vivo experiments. A PVA-C tube with 6 cm in length, 6 mm inner diameter and a wall thickness of 0.25 mm was used. The phantom was connected on one side to an electrical pulse generator and on the other side to a water reservoir to hydrostatically adjust the pressure baseline inside the PVA-C tube (Fig 2A).

As reference standard for global PWV, an axial movable pressure catheter (TSK-Supra, Ebhardt-Söhne GmbH, Geislingen, Germany) was inserted into the PVA-cryogel tube. Fig 2B shows the mounted PVA cryogel phantom with the pressure catheter. The pressure signal was converted into an electrical signal by a pressure transducer (Viggo-Spectramed, Inc., Oxnard, CA, USA) and amplified with a dedicated amplification unit. For the further data processing, the pressure signal was recorded with a sample rate of 2.5 kHz using a commercial storage oscilloscope.

For all PWV measurements, the pulse rate of the electrical pump was adjusted to 1 Hz. All MR experiments were triggered according to the electrical pulse generation signal. Pressure measurements were performed at 16 locations along the pulse wave propagation pathway (mimicking the multi-point TT approach of the MR measurements) with a distance between adjacent measurements sites of 2 mm. All pressure wave acquisitions were averaged 16 times to reduce noise. To estimate the PWV, the same post-processing routines were applied as used for the MR-based multi-point TT method.

### Animal protocol

Sixteen mice were investigated in this study. Four-week-old female ApoE^−/−^ (n = 8) and WT C57Bl/6 (n = 8) mice were obtained from Charles River Laboratories (Sulzfeld, Germany). The ApoE^−/−^ mice (B6.129P2-ApoE^tm1Unc^/J, Stock Number: 002052) had been backcrossed into the C57BL/6J genetic background for at least 10 generations. ApoE^-/-^-mice were fed a high cholesterol diet (TD 88137, ssniff GmbH Soest, Germany), starting at the age of 4 weeks, while the WT mice were fed a regular chow diet. MR imaging was performed at the age of 18 weeks. During the MR experiments, the mice were anesthetized with an isoflurane inhalation (1.5–2.0 Vol.%) applied via a nose cone. The depth of anesthesia was controlled by monitoring of the breathing rate. The temperature of the sample volume inside the RF coil was kept constant at 37°C by adjusting the temperature of the gradient cooling unit. All experimental procedures were in accordance with institutional guidelines and were approved by the Institute of Animal Care of the district government of Lower Franconia.

### In vivo experiments

In order to localize the descending aorta, a set of 2D FLASH experiments was obtained, as previously described [24].

**Fig 3:**
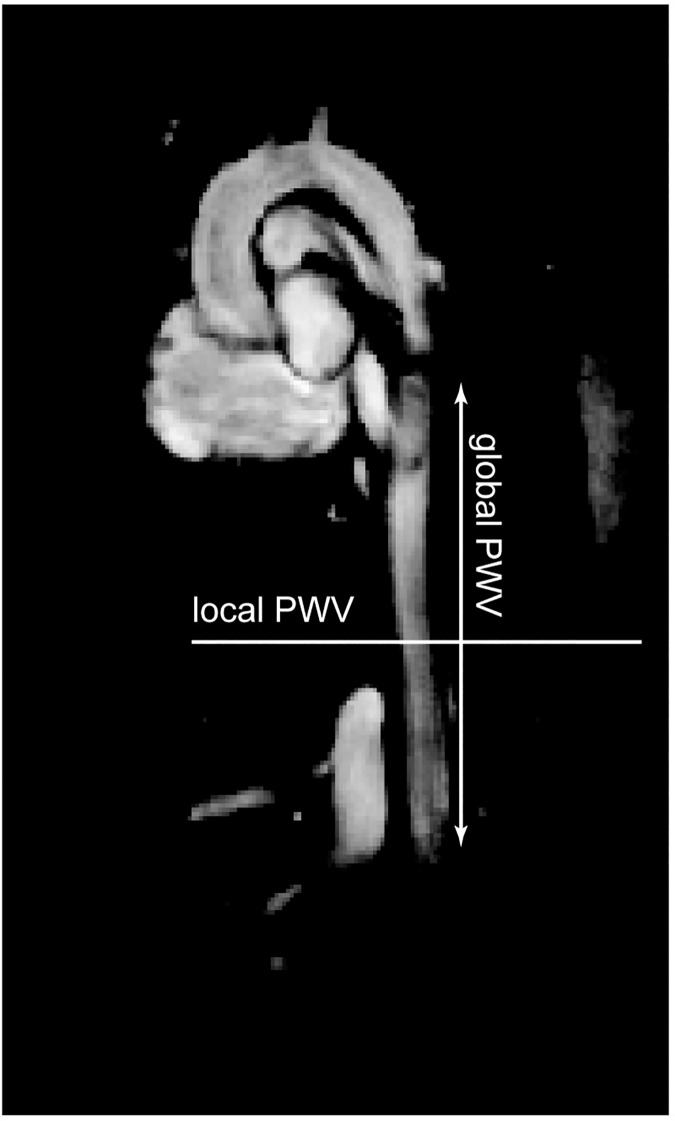
Slice positioning in-vivo. In the abdominal aorta, local PWV was measured in a slice perpendicular to the direction of the vessel. The global PWV was obtained from a slice parallel to the descending aorta.

Global PWV was measured in the descending aorta. To this end, the image slice was aligned with the orientation of the descending aorta. To evaluate the local PWV with the QA method, one image slice was positioned perpendicular to the abdominal aorta at a level ~2 mm below the diaphragm as shown in Fig 3. For both methods, a time window of 40 ms, covering the systolic upstroke, was sufficient to acquire the relevant flow and area information.

The total measurement time for the QA method and the multi-point TT-method respectively was approximately 6 min. Further imaging parameters for the global and the local PWV measurements were: matrix 150 × 150; field of view: global: 3 × 3 cm^2^ / local: 2.2 × 2.2 cm^2^; TE = 1.7 ms; slice thickness: global: 1.4 mm / local: 1.0 mm; flip angle: 40°; total number of time frames: 40.

### Data processing

Basic data processing was performed using customized routines written with MATLAB (The Mathworks Inc., Natick, MA). For morphometric analysis of the vessel geometry, magnitude images were imported into AMIRA (Visage Imaging Inc., San Diego, CA) and segmented manually. Velocity data for the QA- and the TT-method respectively were computed pixel-wise by a linear fit of the phase data as a function of the first moments of the velocity encoding gradients. Using three different encoding steps allowed to estimate the error of the velocity values based on the R^2^ (squared correlation coefficient) calculation. All velocity data points of the segmented areas with R^2^ less than 0.85 were excluded from further calculations.

**Fig 4:**
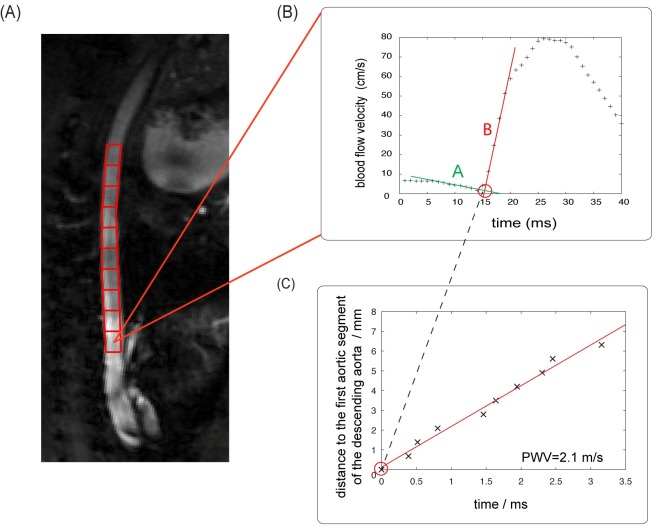
Determination of global PWV. In vivo determination of global pulse wave velocity (PWV) with the multi-point transit-time method in the descending aorta of a wild type mouse (**A-C**). Ten evenly spaced segments for the global PWV measurements (red) and the derived flow curves are shown in a magnitude image of a PC-Cine-FLASH scan (**A**). In each segment (**B**), the arrival time of the pulse wave is determined in the flow curve as the intersection point of the pre-systolic baseline (**A, green**) and the systolic upslope (**B, red**). The slope of the linear fit (**C**) of distance over the according pulse wave arrival times represents the global PWV.

- *TT Method*: To compute the global PWV with the multi-point TT-method, a manually segmented linear section of the descending aorta was divided into 10 (phantom experiments: 20) evenly spaced regions of interests (ROIs). The distance between adjacent ROIs was calculated as the distance between the accordant centers of mass. The longitudinal extension of each ROI was 0.9±0.2 mm (phantom experiments: 1.0 mm). For each segment, the foot of the systolic upstroke was identified as the intersection point of a line fitted to the pre-systolic data points (baseline) and a line fitted to the upslope portion of the early systolic pulse as shown in Fig 4. Flow values greater than 2×standard deviation (SD) of the baseline data points and less than 80% of the peak flow were considered as data belonging to the early systolic upstroke. For the in vivo experiments the mean number of data points for the baseline and the systolic upstroke were 5±2 and 4±1 respectively. The distance from the first ROI was plotted for each subsequent ROI against the accordant arrival time of the systolic pulse wave. PWV was computed as the slope of a line fitted to this plot using a least square fit procedure.

- *QA Method*: The cross-sectional area of the vessel was segmented manually in each time frame during the early systole using the magnitude information of the complex PC-CINE datasets. All segmentations were repeated four times by the same observer and the mean area was calculated for further computations. Blood flow was computed by adding pixel wise all intraluminal velocity values multiplied with the corresponding cross-sectional areas. The start of the systolic flow pulse was identified analogue as in the post-processing for the TT method. Five subsequent data points where taken to evaluate the local pulse wave velocities by fitting a line to the correspondent data points of the QA-plot. Applying a low-pass filter to the data prior to the PWV calculation reduced random high frequency changes of the volume flow- and area-curves.

### Statistical analysis

All data are expressed as mean ± SEM. Statistical analysis was conducted with SPSS software (SPSS Inc, Chicago, IL). Normal distribution of data was confirmed using the Shapiro-Wilk test and homogeneity of variances was tested with Levene's test. Differences between ApoE^-/-^ and WT control mice were tested using an unpaired two-tailed Student`s t-test for normally distributed data and a non-parametric Mann-Whitney-U test for not normally distributed data. A weighted least-squares regression analysis with correction for heteroscedasticity was performed to evaluate the relationship between local and global PWV. A p-value of <0.05 was considered statistically significant and a p-value of <0.01 was considered highly significant.

## Results

### Phantom experiments

**Fig 5:**
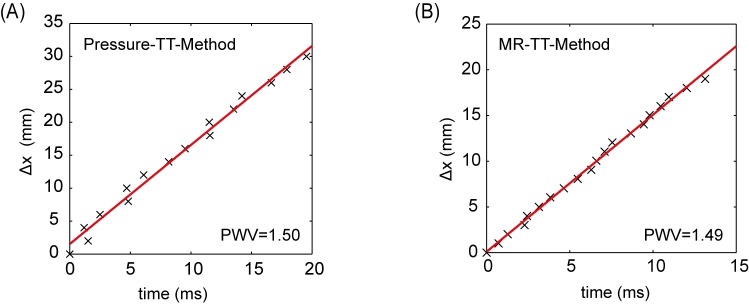
Validation of MR multi-point TT-measurement for global PWV. Direct comparison of the transit time measurements using a pressure sensor (**A**) and the corresponding MR multi-point TT-measurements (**B**) in the PVA-C phantom.

In the vessel phantom, the pressure wire-based and the MR-TT method-based measurements of global PWV were repeated five times. In addition, the MR-based local PWV was determined at the center and two additional sites, distributed equidistantly around the center (Δx = 1 cm) of the vessel phantom. The maximum pulse pressure generated by the electrical pump was 0.5±0.1 kPa. The resulting maximum change of the vessel wall radius was 20±5% between maximum and minimum pulse pressure. Fig 5A shows the spatial position of the pressure wire at different measurement sites plotted against the according arriving times of the pulse pressure wave. The slope of the plot represents a value for the pulse wave velocity. Five measurements with the pressure TT-method resulted in a mean PWV of 1.51±0.03 m/s. Fig 5B shows a representative plot of the global MR-TT-measurement in the vessel phantom. The MR-based mean global PWV for the same five repeated measurements was 1.51±0.04 m/s, thereby showing an excellent agreement with the pressure wire measurement.

The MR-based local PWV measurements were also in good compliance with the pressure wire based global PWV. The mean values for the MR-based local PWV at the three different measurement sites were (from bottom to top): 1.57±0.07 m/s, 1.55±0.09 m/s and 1.51±0.10 m/s. The results of all the validation experiment are summarized in Table 1.

**Table 1:** Results of the validation experiments.

	TT-Press. (m/s)	TT-MR (m/s)	QA-MR 1 (m/s)	QA-MR 2 (m/s)	QA-MR 3 (m/s)
PWV: n = 1	1.58	1.43	1.62	1.41	1.47
PWV: n = 2	1.43	1.56	1.34	1.58	1.81
PWV: n = 3	1.47	1.62	1.21	1.62	1.51
PWV: n = 4	1.49	1.43	1.77	1.32	1.45
PWV: n = 5	1.60	1.50	1.61	1.81	1.59
Mean ± SEM	1.51±0.03	1.51±0.04	1.51±0.10	1.55±0.09	1.57±0.07
P-Value*		0.91	0.97	0.72	0.50

Values of the PWV measured by MRI are in a good agreement with the PWV values obtained by examining transit time of the pressure pulse. Each experiment was repeated five times (n = number of repetition). The results of all measurements and the mean ± SEM are given in the table.

*The differences of means of the MR-samples were tested against the TT-Pressure samples with a two sided T-Test. Abbreviations: MR, magnetic resonance; PWV, pulse wave velocity; QA-MR, PWV measured by flow/area MR method; TT-MR, PWV measured by transit-time MR method; TT-Press, PWV measured by pressure catheter.

### In vivo study

The global PWV in the descending aorta was significantly higher in ApoE^-/-^ mice compared with WT mice (ApoE^−/−^ 2.7±0.2 vs WT 2.1±0.2 m/s, P = 0.023), implying a significant increase in global aortic stiffness due to initial atherosclerotic lesions.

Corresponding to the data from the global PWV, local PWV in the upper abdominal aorta was also significantly elevated in ApoE^-/-^ mice compared with the age-matched control group (ApoE^−/−^ 2.9±0.2 vs WT 2.2±0.2 m/s, P = 0.028).

**Fig 6:**
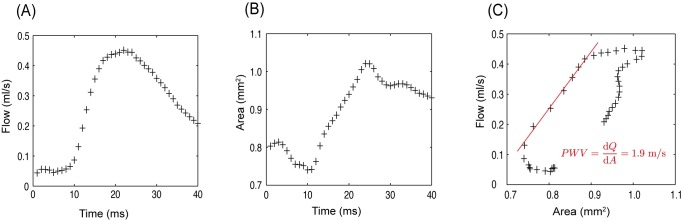
Determination of local PWV. Local PWV is assessed by simultaneously recording flow (**A**) and cross-sectional area (**B**) through an imaging slice perpendicular to the upper abdominal. The slope of the flow/area plot (**C**) represents the local PWV at the examined location (exemplarily dataset shown for a WT mouse).

Fig 6A shows a representative time course of the blood volume flow in the abdominal aorta in a WT mouse and the corresponding cross-sectional area changes (Fig 6B). When plotting cross-sectional areas versus flow-data, the linear relationship in early systole represents the local PWV (Fig 6C).

**Fig 7:**
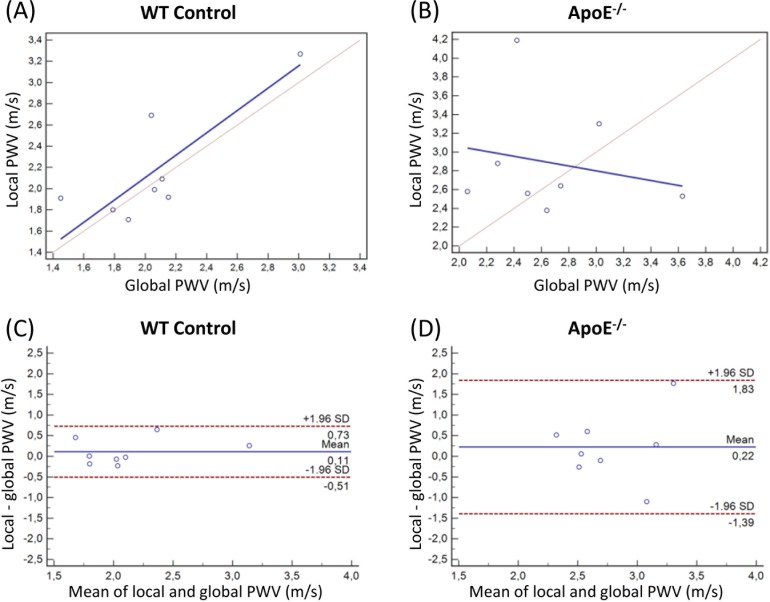
Correlation and agreement between global and local PWV in ApoE^-/-^ and WT mice. Linear regression between global PWV values obtained with the multi-point TT-method and the local PWV, assessed with the QA-method for the WT group (**A**) and the ApoE^-/-^ group (**B,** blue: regression line, red: identity line). The Bland-Altman plots show that there is a higher agreement between local and global PWV in WT mice (**C**) as compared to ApoE^-/-^ mice (**D**).

Within the ApoE^-/-^ as well as the control group, no significant difference for the mean values between local and global PWV values was found. A regression analysis comparing global and local PWV values within the individual groups revealed a highly significant linear relation between local and global PWV in WT animals (R^2^ = 0.75, P<0.01) as shown in Fig 7A. The strong correlation between local and global PWV in WT mice implies an even distribution of the vessel wall elasticity in healthy vessels. In ApoE^-/-^ mice, however, no correlation (R^2^ = 0.07, P = 0.53) between local and global PWV in the individual mice could be found, which implies an inhomogeneous distribution of early vascular stiffening caused by initial atherosclerotic lesions (Fig 7B). The significantly better agreement between local and global PWV becomes also evident by comparing the Bland-Altman plots of global and local PWV in WT (Fig 7C) and ApoE^-/-^ mice (Fig 7D).

## Discussion

This work investigated the relation between global and local PWV in early murine atherosclerosis using ultrahigh field MR microscopy. For the measurements of global PWV, a new MR-based TT-method was introduced and validated in phantom measurements. The measurements in a PVA-C phantom revealed an excellent agreement between the global PWV measured with the MR-based TT-method and the values determined with the pressure wire measurement as reference standard. In addition, the MR-based measurements of local PWV were also in a good agreement with the pressure wire measurements, as expected in a uniform phantom.

In vivo data acquired with the new TT-method resembles well global PWV values obtained with a conventional two-point MR-TT measurement in five-months-old ApoE^-/-^-and C57BL/6J-mice [19]. An essential advantage of the new multi-point TT-method upon a two-point TT method using through plane velocity encoding is the acquisition of just one flow encoded image slice instead of two. In this matter, the reduced measurement time allows for an increased stability of the heart rate during data acquisition, which is the most crucial physiological parameter for an accurate PWV measurement. Furthermore, determination of the onset of the flow pulse at multiple instead of just two sites along the pulse wave propagation pathway reduces the statistical error of the PWV calculation. Since flow data for each ROI is calculated by averaging the correspondent velocity information of each pixel, increasing the number of ROIs decreases the statistical error of determination of the flow data. The determination of the arrival time of the pulse wave is the process generating the most inaccuracy for the PWV calculation. Due to the restricted temporal resolution of 1 ms some kind of interpolation method has to be applied identifying the arrival time at sub-millisecond precision.

Even though the measurement of global PWV, based on MR [25] or doppler ultrasound [26] is widely used in clinical routine and has proven useful in predicting cardiovascular risk [3, 4], it does not provide information on the vascular elasticity at a specific site of the vascular tree. However, in the initial stages of atherosclerosis, local effects like disturbed flow trigger the activation of the proinflammatory transcription factor nuclear factor κB to provoke recruitment of inflammatory cells, leading to impaired vasodilator activity [27].

In contrast to the TT-method, the QA approach enables estimating the local PWV and thus uncovers important insights into the pathophysiology of early atherosclerosis. Our in vivo measurements revealed significantly higher PWV in the ApoE^-/-^ group compared to WT animals, independent from the measurement method. However, a significant difference between diseased and healthy animals becomes evident when evaluating the relation between global and local PWV in individual mice. While a highly significant correlation of global and local PWV values in the WT animals could be found, investigations in ApoE^-/-^ animals showed no significant linear relation between global and local elastic vascular properties. The correlation between global and local PWV as found in the WT group implies a homogeneous elasticity in healthy vessels. The inexistent correlation between local and global PWV in ApoE^-/-^ mice enforces the hypothesis of heterogeneously distributed initial effects of early atherosclerosis on the mechanical properties of the vessel wall. In this view, the generation of an elasticity profile of a vessel by multiple subsequent local PWV measurement may give superior information in terms of characterizing the state of atherosclerosis compared to an averaged information from global PWV measurements. Our results suggest that the heterogeneity of arterial stiffening itself could be a useful marker of early atherosclerosis as compared to absolute local or global PWV values as it has the benefit of compensating for between animal or group differences in blood pressure—on which PWV highly depends. Therefore, future research is needed to investigate the role of elasticity profiles by multiple local PWV measurements in the assessment of early atherosclerosis.

A recent study in a large, adult, multi-ethnic population demonstrated that while age, blood pressure, and smoking are the main determinants of increased aortic stiffness, also the control of cardiovascular risk factors like elevated blood pressure can influence the progression of aortic stiffness[28]. In this study global PWV in the aortic arch was determined with a 10-year follow up, in the light of our results, the use of local PWV at multiple sites may contribute to a faster detection of the impact of therapeutic interventions by revealing early effects on local aortic stiffness.

### Limitations

The study protocol did not include an ex-vivo analysis of aortic elasticity to corroborate the in-vivo measurements. However, all MR-based methods were validated in-vitro in a vessel phantom. The high flip angle of 40° leads to a relatively poor signal intensity of the outer regions of the vessel wall almost comparable to static tissue, hence potentially resulting in an underestimation of the vessel wall boundaries. However, the total loss of SNR by reducing the flip angle was shown to affect the segmentation accuracy significantly in preliminary experiments, exceeding a potentially systematic error due to a blood inflow effect. Apart from that only a heterogeneous baseline shift of the estimation of the cross sectional area during early systole would influence the PWV calculation since the estimation considers only incremental changes of flow and area data.

## Conclusion

We introduced a new MR-based multi-point TT measurement for a rapid and reliable measurement of global PWV, which shows excellent reproducibility compared to the reference standard of a pressure wire and allowed to halve measurement time compared to a conventional two-point TT measurement. Comparative assessment of global and local PWV in ApoE^-/-^ and WT mice revealed that vascular stiffening caused by early atherosclerosis is inhomogeneously distributed over the length of a large vessel. This finding implies that assessing the heterogeneity of arterial stiffness by multiple local PWV measurements may be more sensitive than global measures to identify early atherosclerotic lesions.
